# Anti-tumor antibody profile analysis to harness the potentials of B cells in melanomas and the natural humoral immune response

**DOI:** 10.1186/2051-1426-1-S1-O4

**Published:** 2013-11-07

**Authors:** Beatrix Kotlan, Gabriella Liszkay, Miri Blank, Judit Olasz, Orsolya Csuka, Timea Balatoni, Kinga Borbola, Laszlo Toth, Gyorgy Naszados, Francesco M Marincola, Maria Godeny, Miklos Kasler, Yehuda Shoenfeld

**Affiliations:** 1 Molecular Immunology and Toxicology, National Institute of Oncology, Budapest, Hungary; 2 Oncodermatology, National Institute of Oncology, Budapest, Hungary; 3 Zabludowicz Center for Autoimmune Diseases, Sheba Medical Center, Tel Hashomer, Israel; 4 Pathogenetics, National Institute of Oncology, Budapest, Hungary; 5 Oncosurgery, National Institute of Oncology, Budapest, Hungary; 6 Radiological Diagnostics, National Institute of Oncology, Budapest, Hungary; 7 SIDRA Medical and Research Center, Doha, Qatar; 8 Board of Directors, National Institute of Oncology, Budapest, Hungary; 9 Univ Med Pharm, Tirgu Mures, Romania

## Objectives

Natural humoral immune response and autoimmune mechanisms have great importance in keeping the balance of tumorimmunity, although it has not yet been fully understood. We aimed to reveal potential anti-tumor immune response by immunoglobulin (Ig) profile analysis of patients with metastatic melanomas.

## Methods

A complex panel assay has been performed at expressed DNA and protein levels on antibodies originating from patients' peripheral blood (n = 92) or cancerous tissue biopsies (n= 87) (ETT TUKEB 16462- 02/2010). Heavy and light chain immunoglobulin variable gene regions were sequenced and analysed with Vector NTI Advance 11, Bioedit 7.0, ClustalX2.0.11, TreeView 1.6.6 programs using available databases (IMGT, Blast). Patients' sera, purified human Ig preparations and antibody fragments from tumor infiltrating B cells were tested by ELISA, immunofluorescence FACS and confocal laser microscopy.

## Results

**Figure 1:**
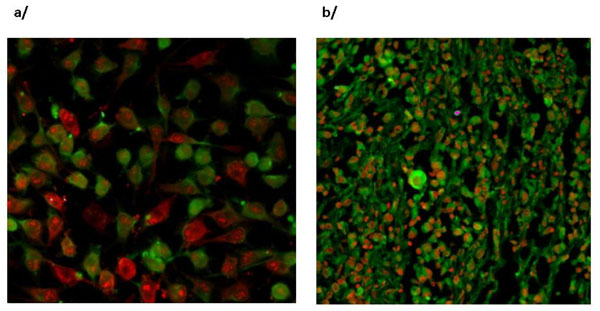
Human purified immunoglobulin of patient (a/) and antibody fragments developed (b/) show cancer associated antigen specific binding on SK-Mel 28 cells and melanoma tissues.

Cluster analysis revealed specific antibody variable region gene subgroups in the VH3 family, amongst which there are the ones with cancer associated antigen binding capacity. The purified immunoglobulin's strong SK-Mel28 melanoma binding potential paralleled with the clinical outcome. Some selected expressed antibody fragments showed tumor associated antigen labelling on melanoma tissues (Fig. 1). A competitive cell membrane ELISA has been standardized for measuring patients' sera in terms of various cancer associated antigen binding capacity. Data are under evaluation concerning their value to predict anti cancer humoral immune response.

## Conclusions

We could prove the extensive presence of highly tumor associated unique GD3 sialilated glycosphingolipid specific antibody variable regions in patients with melanoma. Our novel panel assay confirmed the potentials of antibody profile analysis in characterizing anti tumor humoral immune response and its worth for diagnostics.

